# Avoiding diagnostic errors in psychosomatic medicine: a case series study

**DOI:** 10.1186/s13030-018-0122-3

**Published:** 2018-03-13

**Authors:** Atsuko Koyama, Yoichi Ohtake, Kanae Yasuda, Kiyohiro Sakai, Ryo Sakamoto, Hiromichi Matsuoka, Hirokuni Okumi, Toshiko Yasuda

**Affiliations:** 1 Department of Psychosomatic Medicine, Faculty of Medicine, Kindai University, 377-2, Ohno-higashi, Osaskasayama City, Osaka, 589-8511 Japan; 2 General Internal Medicine, Sakai City Medical Center, 1-1-1 Ebaraji-cho, Nishi-ku Sakai, Osaka, 593-8304 Japan; 3 Department of Psychosomatic Medicine, Sakai Hospital, Kindai University, 2-7-1, Harayamadai, Minami-ku Sakai, Osaka, 590-0132 Japan

**Keywords:** Diagnostic error, Psychosomatic medicine, Psychosomatic disease, Cognitive bias

## Abstract

**Background:**

Non-organic lesions or diseases of unknown origin are sometimes misdiagnosed as “psychogenic” disorders or “psychosomatic” diseases. For the quality of life and safety of patients, recent attention has focused on diagnostic error. The aim of this study was to clarify the factors that affected misdiagnoses in psychosomatic medicine by examining typical cases and to explore strategies that reduce diagnostic errors.

**Case presentation:**

The study period was from January 2001 to August 2017. The data of patients who had visited the Department of Psychosomatic Medicine, Kindai University Hospital and its branches, Sakai Hospital and Nihonbashi Clinic, were collected. All patients were aged 16 years or over. Multiple factors, such as age, sex, presenting symptoms, initial diagnosis, final diagnosis, sources of re-diagnosis and types of diagnostic errors were retrospectively analyzed from the medical charts of 20 patients. Among them, four typical cases can be described as follows. Case 1; a 79-year-old woman, initially diagnosed with psychogenic vomiting due to depression that was changed to gastric torsion as the final diagnosis. Case 2; a 24-year-old man, diagnosed with an eating disorder that was later changed to esophageal achalasia. Case 10; a 60-year-old woman’s diagnosis changed from conversion disorder to localized muscle atrophy. Case 19; a 68-year-old man, appetite loss from depression due to cancer changed to secondary adrenal insufficiency, isolated ACTH deficiency (IAD).

**Conclusion:**

This study showed that multiple factors related to misdiagnoses were combined and had a mutual influence. However, they can be summarized into two important clinical observations, diagnostic system-related problems and provider issues. Provider issues contain mainly cognitive biases such as Anchoring, Availability, Confirmation bias, Delayed diagnosis, and Representativeness. In order to avoid diagnostic errors, both a diagnostic system approach and the reduction of cognitive biases are needed. Psychosomatic medicine doctors should pay more attention to physical symptoms and systemic examination and can play an important role in accepting a perception of patients based on a good, non prejudicial patient/physician relationship.

## Background

Psychosomatic medicine was established in Japan in 1996 as one of the specific medical fields in which “psychosomatic disorders” are dealt with, and it has been developing widely in both Japan and Germany. The department of psychosomatic medicine is classified as part of internal medicine, not psychiatry, and attends to patients complaining of physical symptoms due to psychosocial distress.

Psychosomatic disorders are defined as physical diseases whose onset and course are closely related to psychosocial factors and contain both organic and functional disorders [1]. The diagnosis of true “psychosomatic diseases” should be based on strict differential diagnoses, including functional disorders. However, non-organic lesions or diseases of unknown origin are sometimes diagnosed as “psychogenic” disorders or “psychosomatic” diseases. These problems are becoming more prominent according to an increase in the number of patients with psychosomatic diseases. The reduction of diagnostic error is an important goal for the quality of life and safety of patients. The aim of this study was to clarify the factors that affected misdiagnoses in psychosomatic medicine by examining typical cases and to explore strategies that reduce diagnostic errors.

## Material and methods

### Patients

The inclusion criteria for this study were as follows:

1. Patients who had visited the Department of Psychosomatic Medicine, Kindai University Hospital and its branches, Sakai Hospital and Nihonbashi Clinic, from January 2001 to August 2017.
2. Patients who were considered to have physical symptoms due to psychosocial distress at their first visit.
3. Aged 16 years or over, because our department is associated with internal medicine and only provides care for those of high school age or older.

The exclusion criteria were as follows:

1. Patients with primary psychiatric diseases, for example having hallucination and/or delusion.
2. Patients who were unwilling to participate and contacted us to refuse participation.

### Design and setting

This was planned as a case series study.

All patients who visited our department for the first time filled out a systemic medical questionnaire including their demographic background, subjective physical complaints, and psychological distress, after which semi-structured interviews were performed by doctors.

During the study period, all items assessed during routine clinical practice were extracted from the patients’ medical charts. Multiple factors, such as age, sex, presenting symptoms, initial diagnosis, final diagnosis, sources of re-diagnosis, types of diagnostic errors, and the interval between an initial diagnosis and a final diagnosis were retrospectively analyzed from the medical charts of 20 patients. The interval was classified into six categories; less than 1 month, 1 to 3 months, 3 to 6 months, 6 to 12 months, 12 to 24 months, and more than 24 months.

The co-authors gathered cases in which the final diagnosis was significantly different than the initial diagnosis. Almost all cases, except for cases 3 and 20, were referred from another hospital or another department in Kindai University Hospital or Sakai Hospital that reported their initial diagnosis. As for cases 3 and 20, both the initial and final diagnosis were made in our department. Each main doctor in the cases made a true final diagnosis through systematic examination, but in some cases a heuristic method helped determine the diseases. Two doctors independently examined the process of diagnosis and judged the type of diagnostic error. After discussing their findings, their consensus on the type of diagnostic error was adopted.

## Results

### Demographic and clinical characteristics

Approximately 2200 new patients come to Kindai University Hospital and its branches, Sakai Hospital and Nihonbashi Clinic, every month, with 20 to 25 of them visiting the Department of Psychosomatic Medicine. Twenty cases were eligible for this study according to the inclusion criteria.

Detailed patient characteristics are listed in Table 1, which contains the case number, age, sex, symptoms, initial diagnosis, final diagnosis, sources of re-diagnosis, types of diagnostic errors, and the interval between the initial diagnosis and the final diagnosis. The age ranged from 24 to 81 years and the sex ratio was 14 male to 6 female. In comparison, the sex ratio of the patients in our department is 1 male to 2.5~ 3 female.

**Table 1:**
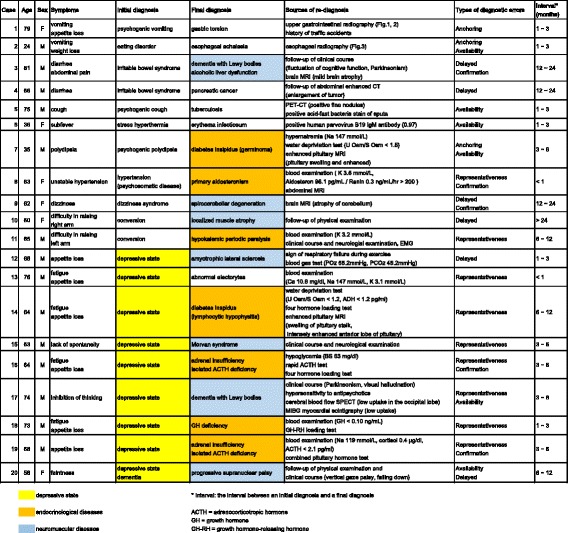
Patient Characteristics

As an initial diagnosis, three cases were diagnosed with “psychogenic” diseases and six with “psychosomatic” diseases. In addition, two cases were diagnosed with conversion disorder and nine with depressive state. As for the final diagnosis, seven cases were identified by blood examination and detailed hormonal test as having endocrinologic diseases. Another seven cases were diagnosed with neurological diseases after follow-up of the clinical course and brain MRI. Adequate examinations were effective in making the true diagnoses. For example, radiography instead of endoscopy in cases 1 and 2, targeted examination such as the acid-fast bacteria stain in case 4, and an antibody test in case 6.

The interval between the initial diagnosis and the final diagnosis ranged from less than 1 month to more than 24 months. The most difficult case in this study was case 10.

## Case presentation

There were several types of diagnostic errors and differences in the clinical courses. Among the twenty cases, four typical, characteristic cases that are educational and that contain the five representative types of diagnostic errors identified in this study will be described in detail. These cases show give a number of lessons as to the path taken from an initial misdiagnosis to a final, true diagnosis.

### Case1

#### Initial diagnosis: Psychogenic vomiting due to depression

A 79-year-old, previously healthy woman presents with symptoms of vomiting and appetite loss. Nine months previously she was hit by a truck and had a blood pneumothorax, a right clavicle fracture, and left rib fracture. She completely recuperated in three months. During the long-term admission she lost confidence in her recovery and when she returned home she sometimes felt anxiety about living alone. Two months before coming to our department she began to vomit and gradually fell into a depressive mood and suffered appetite loss. She refrained from eating food in fear of vomiting, and thus lost weight. An upper gastrointestinal endoscope examination at another clinic revealed a normal limit, and she was referred to our department with a diagnosis of psychogenic vomiting or eating disorder due to depression.

**Fig. 1:**
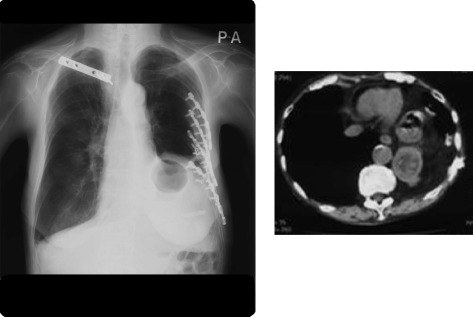
Chest X-ray and chest CT of case 1 Chest X-ray (left) and chest CT (right) show a left diaphragm hernia. The left diaphragm deviates upwards into the thoracic cavity and gastric gas is seen in the gastric body

**Fig. 2:**
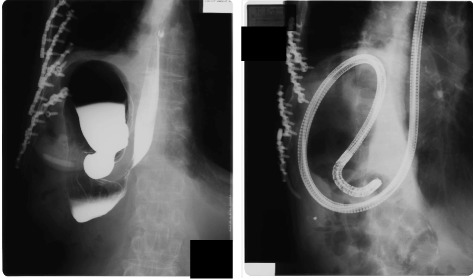
The upper gastrointestinal radiography and the upper gastrointestinal endoscope examination of case 1 The upper gastrointestinal radiography (left) shows gastric torsion with an adhesion of the gastric body in the upper side rather than the gastric fornix. The upper gastrointestinal endoscope examination (right) shows the torsional axis and position of the gastric body

Chest X-ray and chest CT (Fig. 1) revealed a left diaphragm hernia, and upper gastrointestinal radiography showed gastric torsion with the adhesion of gastric body in the upper side rather than gastric fornix (Fig. 2). The upper gastrointestinal endoscope examination showed almost normal mucosa, although it was slightly reddish and edematous. The reset of gastric position by gastrointestinal endoscope was impossible due to tight adhesion of the gastric body and left diaphragm and also impossible due to gastric fistula. A radical operation for the diaphragm hernia was performed and the gastric axis and position were normalized. After the operation, she recovered her appetite without vomiting and was discharged on the 23rd day.

**Final diagnosis: gastric torsion.**

### Case 2

#### Initial diagnosis: Eating disorder

A 24-year-old man presents with symptoms of nausea, vomiting, and weight loss. During the previous year, he sometimes suffered from nausea and vomiting and lost 15 kg (82 → 67 kg) in spite of having a normal appetite. His height was 170.7 cm. At first, he desired weight loss because of his obese body image. He mentioned that his ideal body weight was 65 kg. An upper gastrointestinal endoscope examination at another clinic revealed a normal limit and medicine for gastrointestinal movement did not improve his symptoms. He was referred to our department with a diagnosis of an eating disorder.

**Fig. 3:**
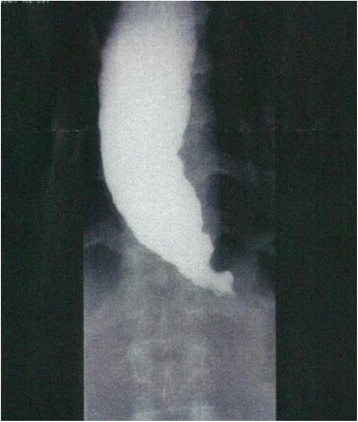
The esophageal radiography of case 2. The esophageal radiography shows dilation of the esophagus and a “bird-beak” appearance, which are characteristics of esophageal achalasia

Under observation after admission, he sometimes vomited unexpectedly and at other times swallowed food successfully, so it was not self-induced vomiting. The esophageal radiography showed esophageal achalasia (Fig. 3). Transendoscopic myotomy mitigated his symptoms.

**Final diagnosis: esophageal achalasia.**

### Case 10

#### Initial diagnosis: Conversion disorder

A 60-year-old woman presented with difficulty in raising her right arm. For the previous year she had felt weakness and numbness in her right arm. During that time, she moved to her son’s home and lost contact with her old friends. Her relationship with her son’s wife was worsening and she avoided meals that her son’s wife cooked. A specific examination at the department of neurology revealed no abnormality. She was referred to our department with a diagnosis of conversion disorder.

The symptom was gradually progressive and sometimes improved, but at one point improved and worsened on the same day. Several months before the final diagnosis the symptom progressed markedly. When she complained of slight back pain and weakness, we examined her whole body again and found localized muscle atrophy in the right area of her back. We consulted the neurologist again and she was diagnosed with localized muscle atrophy after an electromyogram and muscle biopsy. There is no radical treatment for this problem, so she received physical therapy and was kept under observation.

**Final diagnosis: localized muscle atrophy.**

### Case 19

#### Initial diagnosis: Depression due to cancer (appetite loss)

A 68-year-old man presented with symptoms of appetite and weight loss. He suffered from gastric cancer and had had a partial gastrectomy three years previously. He recovered from it and his activity of daily living was normal. However, over the three months before reporting to the hospital, after suffering a common cold he gradually lost his appetite and weight (from 58 to 48 kg). He could consume only semisolid food, such as tofu, and a small amount of water, and stayed in bed almost all day long. Upper gastrointestinal endoscope examination, abdominal CT, and tumor marker were normal at the department of gastroenterology. Medicine for gastrointestinal movement did not improve his symptoms. He was referred to our department with a diagnosis of depression due to cancer.

His general condition gradually worsened and he was finally admitted due to dehydration. On the 1st hospital day, serum Na was 135 mmol/L due to dehydration. After drip infusion and tubal feeding for several days, it was found that serum Na was very low (119 mmol/L). In the next step, endocrinological laboratory studies showed very low serum and plasma levels of adrenocorticotropic hormone (ACTH) (< 2.1 pg/ml) and cortisol (0.4 μg/dl). Following dynamic tests for pituitary hormone secretion in response to combined stimulation with corticotropin-releasing hormone (CRH), thyrotropin-releasing hormone (TRH), luteinizing hormone-releasing hormone (LH-RH), and growth hormone-releasing hormone (GH-RH) revealed a blunted response of ACTH. Thus, the patient was diagnosed with isolated ACTH deficiency (IAD). The replacement of hydrocortison 15-20 mg/day improved his condition, he gradually became able to have meals, and he was finally discharged.

**Final diagnosis: secondary adrenal insufficiency, isolated ACTH deficiency (IAD).**

## Discussion

To summarize the characteristics of the 20 patients, their ages ranged from 24 to 81 years, which seems rather high considering that our department is associated with internal medicine and our hospital only treats patients aged 16 years and over. No age-related factors were found in this study; however, older patients might have a tendency to suffer from complication of multiple diseases due to aging, which makes it difficult to reach a true simple diagnosis.

As for sex-specific features, the reason why there were more male than female patients in this study is unknown. However, the difference in prevalence by sex might give an important hint for reconsidering the diagnostic process. For example, despite the incidence of eating disorder being much higher in female patients, the patient in case 2 was a young male, which led to a more prudent clinical assessment. Therefore, it is important to consider the prevalence of diseases as they relate to age and sex in order to make a correct diagnosis.

This study shows that multiple factors related to misdiagnosis were combined and had a mutual influence. However, they can be summarized into two important clinical observations, diagnostic system-related problems and provider issues [2–5].

### Cause of diagnostic errors

#### Diagnostic system-related problems

Previous studies showed that clinical reasoning is based on two systems. System 1 is an intuitive process based on heuristics [6, 7], and System 2 is an analytical process. Each system has both advantages and disadvantages, as summarized in Table 2. Both systems can interact and/or override each other [8]. Although System 1 seems to be prone to failure, it has been verified that the two processes are equally effective [9].

**Table 2:** Comparison of System 1 and System 2: Types of clinical reasoning

	Intuitive process	Analytical process
	System 1	System 2
Examples	Heuristics	Algorithm
	Pattern recognition	Hypothetical-deductive
Feature	Snapshot diagnosis	Comprehensive diagnosis
	Unconscientious	Conscientious
Advantages	Faster	Scientific
	Efficient	Analytical
Disadvantages	Biases	Slower

In this study, both systems were used, however, the doctors ranged from a novice who preferred System 2 to an expert who is good at System 1, and the interaction of both systems might not have functioned effectively.

#### Provider issues

Provider issues contain mainly cognitive biases [3–5], failures in perception and failed heuristics due to lack of medical knowledge and/or clinical experience [5, 10]. Previous studies described several types of cognitive biases, such as Anchoring, Availability, Confirmation bias, Delayed diagnosis, and Representativeness [5, 8, 11, 12], as in Table 1.

Anchoring is the tendency to be affected by an initial impression that is not adjusted by later information. This was seen in cases 1, 2, and 7.

Availability is the tendency to judge diagnosis by recent experience and the memory of diseases. This was seen in cases 2, 5, 6, 7, 17, 20.

Confirmation bias is the tendency to seek data/evidence to support a diagnosis and to cast away data/evidence that refutes it. This was seen in cases 3, 8, 9, 16, and19.

Delayed diagnosis means that long-term observation is necessary until a true diagnosis is made. This was seen in cases 3, 4, 9, 10, 12, and 20.

Representativeness is the tendency to look for prototypical manifestations of diseases and to miss atypical variants. This was seen in cases 8, 11, and 13–19.

### Strategies to reduce diagnostic errors

Based on this study, several measures were found that would reduce diagnostic errors.

#### Diagnostic system improvement

First, There are several proposals for system level improvement, for example using checklists [13, 14] and ‘12 tips’ [15]. Moreover, interventions that use decision-making skills, such as computer searching engines, diagnostic tools on the internet, and facilitating access to information, second opinions, and specialists might be useful [10].

#### Provider issues

Next, several issues were found in this study at the individual level.

1. Doctors can never make a true diagnosis of diseases that do not come to mind, for example the endocrine disorders in cases 14–19. Even if incidence and prevalence are very low [16], clinicians should take into account the possibility of rare diseases and their characteristics, such as the IAD in cases 16 and 19. Targeted hormonal examinations are essential for diagnosing ACTH deficiency, severe growth hormone hypoplasia, and diabetes insipidus by lymphocytic hypophysitis. For this issue, the improvement of medical knowledge and diagnostic skill through systematic medical education is essential [8].
2. It is important that trivial abnormal findings should not be ignored. Detailed analysis of examination data is also necessary, as seen in cases 5–9, 11, 13, 14, 16, 18, and 19.
3. Long term observation is necessary to make a true diagnosis [14], for example, neuromuscular diseases, such as the localized muscular atrophy and amyotrophic lateral sclerosis of cases 3, 4, 9, 10, 12, and 20. This is related to Delayed diagnosis. The most difficult case in this study was case 10, and the time it took to reach a final, correct diagnosis was more than 24 months. The initial specific examination in the Department of Neurology revealed no abnormality and muscle atrophy was not found, therefore it took a long time until the symptoms progressed markedly and muscle atrophy became obvious.
4. Easy judgement of “psychogenic” diseases and “depressive state” should be avoided, as in cases 1, 5, 6, 7, and10–19. For example, IAD can mimic depressive disorder due to the symptoms of appetite loss and hypoactivity, as in cases 16 and 19 [17]. Several previous studies suggest that misdiagnosis of depression in primary care outpatients occurs fairly often, and even in educational general hospitals non-psychiatric house staff frequently misdiagnose psychiatric disorders [18]. This can be improved by psychiatric training/education [19, 20] and using the screening instruments mentioned previously in I. Diagnostic system improvement.
5. If an organic disorder is excluded, there is a possibility of a functional disorder. In cases 1 and 2 the mucosal surface was intact during endoscopic examination, however, there was a functional disorder, such as esophageal achalasia. The definition of psychosomatic diseases contains both organic and functional physical disorders, such as functional somatic syndrome, functional dyspepsia and irritable bowel syndrome. It is the principle of the diagnosis of psychosomatic diseases.
6. Physician overconfidence [21] and emotional reactions to patients [22] lead to misdiagnosis. Information from former doctors should be verified, without prejudice, as in cases 1 and 2. The correction of cognition bias by Availability is needed. It is important to include the patient perspective [23] and for patients to be “co-producers” in making a safer diagnostic process [23]. Previous studies showed that a semi-structured interview and the use of positive criteria are effective [24]. Building a good relationship between doctors and patients and cultivating the therapeutic self and self-esteem are also fundamental in psychosomatic medicine.

### Limitations

The present study has several limitations.

First, this study was based on consultation cases only in our hospital. Further studies are needed to clarify factors related to the misdiagnosis of patients visiting departments of psychosomatic medicine throughout Japan.

Second, there is difficulty in discerning the exact factors of misdiagnosis. Clinical reasoning is based on multiple aspects and it is impossible that all clinicians in this study could recall the exact situations and the cause of diagnosis due to time overlay and differences in their clinical knowledge.

Third, there is the inevitable bias of any retrospective analysis in which the outcome is known.

Although our study has several limitations, some highly suggestive results can be regarded as helpful information for clinical psychosomatic practice and for identifying topics for future studies. In order to elucidate the causes of diagnostic errors and to improve strategies to promote psychosomatic medicine, further research addressing the present study’s limitations is necessary.

## Conclusion

There is a high possibility of misdiagnosis among patients diagnosed with “psychogenic” disorders or “psychosomatic” diseases. In order to avoid diagnostic errors, both a diagnostic system approach and the reduction of cognitive biases are needed. Psychosomatic medicine doctors should pay additional attention to physical symptoms and systemic examination and can play an important role by adopting a perception of patients based on a good, non-prejudicial patient/physician relationship.

## Abbreviations

ACTH: Adrenocorticotropic hormone
CRH: Corticotropin-releasing hormone
GH-RH: Growth hormone-releasing hormone
IAD: Isolated ACTH deficiency
LH-RH: Luteinizing hormone-releasing hormone
TRH: Thyrotropin-releasing hormone
